# Modified overtube insertion technique to facilitate safe endoscopic cricopharyngeal myotomy for management of Zenker’s diverticulum

**DOI:** 10.1055/a-2436-1113

**Published:** 2024-11-29

**Authors:** Tony He, Sunil Gupta, Katarzyna M. Pawlak, Natalia Soledad Causada Calo, Jeffrey D. Mosko, Christopher Teshima, Gary May

**Affiliations:** 1 10071 Advanced Therapeutic Endoscopy, St Michael’s Hospital, Toronto, Canada; 2 Gastroenterology, St. Vincent’s Hospital, Melbourne, Australia; 3 8539 Gastroenterology and Hepatology, Westmead Hospital, Sydney, Australia

A 73-year-old woman with a history of progressively worsening dysphagia presented with the inability to tolerate solids or liquids. In the past month, she had lost 10 pounds (4.5 kg) of weight. Gastroscopy revealed a 2-cm Zenker’s diverticulum, located at 19 to 21 cm from the incisors and centered on the 9 o’clock position. Residual solid food was visualized within the diverticulum ( Fig. 1 ). We proceeded to perform an endoscopic Zenker’s diverticulotomy ( Video 1 ). Peroral endoscopic myotomy was not considered because of the short nature of the diverticulum.

**Fig. 1:**
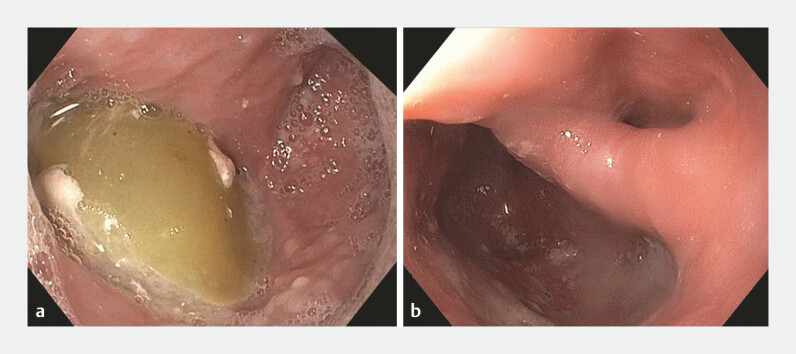
Endoscopic diagnosis of Zenker’s diverticulum. **a** Residual food within the diverticulum. **b** Diverticulum after endoscopic cleaning.

Endoscopic Zenker’s diverticulotomy performed for a symptomatic 2-cm Zenker’s diverticulum using a modified overtube insertion technique.Video 1

We first cleaned the diverticulum and then placed a 0.035-inch guidewire into the stomach. We used a Zenker’s diverticulum overtube (ZDO-22-30; Cook Medical, Bloomington, USA). The 40-mm length of the ZDO distal flap is designed to protect the anterior esophageal wall, whilst the 30-mm length distal flap protects the posterior diverticular wall. To help facilitate safe insertion of the ZDO we modify it by creating a hole using a 22G needle at the apex of the 40-mm length distal flap ( Fig. 2 **a, b** ). The guidewire is then passed through the hole, allowing wire-guided insertion of the ZDO ( Fig. 2 **c, d** ). Once placed correctly, the ZDO isolates the muscular septum ( Fig. 3 ).

**Fig. 2:**
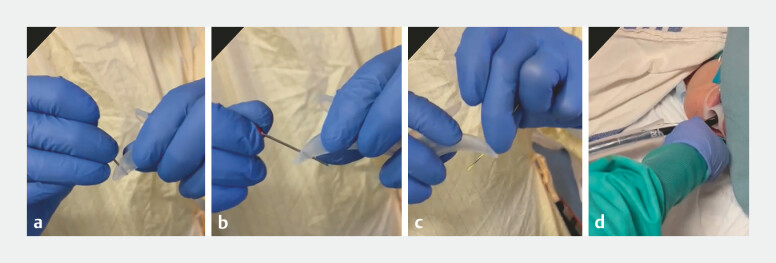
**a, b** Using a 22G needle, a hole is created at the apex of the 40-mm length distal flap. **c** The 0.035-inch guidewire is inserted through the hole. **d** Wire-guided insertion of the Zenker’s overtube towards the diverticulum.

**Fig. 3:**
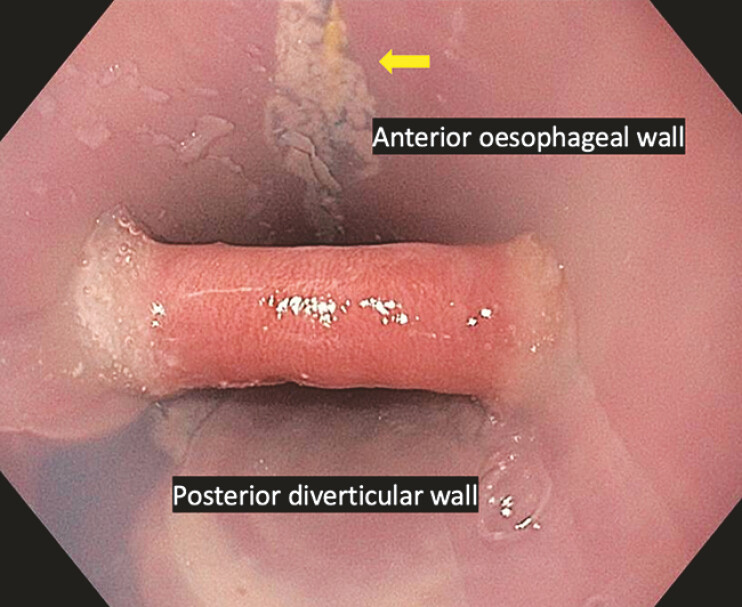
The Zenker’s overtube successfully isolates the muscle septum. The 0.035-inch guidewire can be seen along the anterior esophageal wall (yellow arrow).

A complete myotomy of the cricopharyngeal muscle was then performed with the DualKnife J (Olympus, Tokyo, Japan). The myotomy defect was closed with 6 through-the-scope metal clips. Endoscopic evaluation post clip closure revealed complete flattening of the diverticulum and easy passage of the gastroscope into the esophagus. Within a few days the patient’s dysphagia for solids and liquids completely resolved.

Freehand or cap-assisted endoscopic Zenker’s diverticulotomy can be technically challenging. The anatomical position of the diverticulum can result in endoscope instability and unstable endoscopic views. A ZDO can be placed to safely expose, stretch, and fix the septum, whilst also protecting the anterior esophageal wall and posterior diverticular wall. This allows a safer and more stable endoscopic myotomy 1 2 . ZDO placement however can be difficult, even with endoscopic guidance. We present a simple modification to the ZDO that we have used for more than 5 years that facilitates an easier, more accurate, and less traumatic insertion.

Endoscopy_UCTN_Code_TTT_1AO_2AP

## Notes

Endoscopy E-Videos https://eref.thieme.de/e-videos
*E-Videos* is an open access online section of the journal *Endoscopy* , reporting on interesting cases and new techniques in gastroenterological endoscopy. All papers include a high-quality video and are published with a Creative Commons CC-BY license. Endoscopy E-Videos qualify for HINARI discounts and waivers and eligibility is automatically checked during the submission process. We grant 100% waivers to articles whose corresponding authors are based in Group A countries and 50% waivers to those who are based in Group B countries as classified by Research4Life (see: https://www.research4life.org/access/eligibility/ ). This section has its own submission website at https://mc.manuscriptcentral.com/e-videos .
